# The Currency

**Published:** 1878-01

**Authors:** 

**Affiliations:** Horseheads


### THE CURRENCY.

FARMER.

In 1865 we had a total circulation of $900,000,000 currency in round numbers. This amount includes 5 and 6 per cent. legal tenders, postal currency, and National Bank note circulation.

January 1st, 1878, we have a total circulation of about $700,000,000 in legal tender National Bank notes, and $30,000,000 in silver, which has taken the place of a like amount of postal currency which has been redeemed in silver by the Government within the last two years—-showing a contraction of currency amounting to $200,000,000.

The value of the products of the country and of all property offered in the market for sale, is governed by the amount of money in circulation with which to purchase the same. Thus, the withdrawal of two-ninths of the money in the land from circulation, since 1865, of itself reduces, in general, the value of all merchandise and property in the same or a greater proportion ; and, added to that, an extraordinary depression of all business interests in the country, caused by violent fluctuations of values and a growing disposition, with every one, to sell whatever has been steadily declining in value for the past four years, are adequate reasons for the present stagnation of business.

The value of an irredeemable paper currency depends upon its amount, and, as you decrease the amount, you increase its purchasing power. For example: in 1865 wheat was worth two dollars per bushel ; therefore $1,000 of the $900,000,000 currency then in circulation, would purchase five hundred bushels of wheat. At the present time, the same wheat is worth $1.40 per bushel ; $1,000 of the present currency in circulation ($700,000,000) will purchase 741 bushels of wheat, with an unusual foreign demand, which will balance the extraordinary crop of 1877, while the same large increase in the purchasing power of money as applied to wheat, extends to every staple product in the land. Follow this proportion one step further : the farmer who borrowed $1,000 in 1865, would be obliged now to repay the same in a currency, the relative value of which would be as $1,428 to $1,000.

The farmer who in 1865, with the same crop of wheat would have been enabled to pay his indebtedness out of the avails and would have had a surplus of $428 to expend with the merchants and manufacturers, or have employed additional labor on his farm to that amount, now has no surplus to spend, and the effect is everywhere apparent by the prostrate condition of all branches of business.

The re-monetization of silver by Congress, would produce an immediate resumption of specie payments, add to our currency at once $200,000,000, which is now used as merchandise, and acts no part of money. It would also stimulate every industry of the land and ensure a safe and speedy return to business prosperity.

That silver should at once be re-monetized, as a sound business proposition, no one can deny. The Government, like individuals, is largely in debt, and $1,600,000,000 of national indebtedness is payable, by its terms, in coin, and the value of that coin is specified, that it might not be debased or depreciated in value, gold 25 $\frac{8}{100}$ grains, silver 412½ grains fine.

By examining the production in the United States of the two metals, you will find that in 1873 the production of gold was $36,000,000 and silver $36,500,000, with silver increasing largely over gold each year. In view of the above facts, was it not folly, to use a mild term, for Congress in 1873 to cut off the strongest leg of our financial system and cripple the ability of this nation to pay its indebtedness, by making our debt payable, at once, in a currency more scarce and difficult to obtain ?

The reasons for Congress passing the demonetization bill have never been made public, but it is strongly hinted that $3,000,000 in money was influentially brought to bear upon the question, under the management of one Seberti in the interest of the foreign bondholders ; and it was a bargain for them, as it at once enhanced the value of the securities $160,000,000, or ten per cent. and added that amount virtually, to our indebtedness.

The interests of the debtor and the creditor should not be antagonistic, but when the holders of national securities attempt to obtain the payment of such indebtedness in gold alone, the government is in duty bound to protect the tax payers, not at the expense or detriment of the bondholders, but according to the terms of the contract under which they were issued, and not increase the burdens of an over taxed people.

HORSEHEADS, DEC. 20th 1877.

